# Speciation‐by‐depth on coral reefs: Sympatric divergence with gene flow or cryptic transient isolation?

**DOI:** 10.1111/jeb.13731

**Published:** 2020-11-20

**Authors:** Carlos Prada, Michael E. Hellberg

**Affiliations:** ^1^ Department of Biological Sciences University of Rhode Island Kingston RI USA; ^2^ Department of Biological Sciences Louisiana State University Baton Rouge LA USA

**Keywords:** depth, gene flow, historical demography, speciation, sympatry, transient isolation

## Abstract

The distributions of many sister species in the sea overlap geographically but are partitioned along depth gradients. The genetic changes leading to depth segregation may evolve in geographic isolation as a prerequisite to coexistence or may emerge during primary divergence leading to new species. These alternatives can now be distinguished via the power endowed by the thousands of scorable loci provided by second‐generation sequence data. Here, we revisit the case of two depth‐segregated, genetically isolated ecotypes of the nominal Caribbean candelabrum coral *Eunicea flexuosa*. Previous analyses based on a handful of markers could not distinguish between models of genetic exchange after a period of isolation (consistent with secondary contact) and divergence with gene flow (consistent with primary divergence). Analyses of the history of isolation, genetic exchange and population size based on 15,640 new SNP markers derived from RNAseq data best support models where divergence began 800K BP and include epochs of divergence with gene flow, but with an intermediate period of transient isolation. Results also supported the previous conclusion that recent exchange between the ecotypes occurs asymmetrically from the Shallow lineage to the Deep. Parallel analyses of data from two other corals with depth‐segregated populations (*Agaricia fragilis* and *Pocillopora damicornis*) suggest divergence leading to depth‐segregated populations may begin with a period of symmetric exchange, but that an epoch of population isolation precedes more complete isolation marked by asymmetric introgression. Thus, while divergence‐with‐gene flow may account for much of the differentiation that separates closely related, depth‐segregated species, it remains to be seen whether any critical steps in the speciation process only occur when populations are isolated.

## Graphical abstract

Two depth‐segregated, genetically isolated ecotypes of the sea fan *Eunicea flexuosa* co‐occur across the Caribbean, with high levels of gene flow among populations. Demographic analyses based on SNPs derived from RNA‐seq data suggest a long history of genetic exchange during this pairs' divergence, but also 100K years of transient isolation that may have played a key role in finalizing speciation.

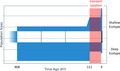

## 1 INTRODUCTION

Most multi‐cellular marine organisms found in shallow temperate or tropical waters possess a pelagic larval stage in their life cycle (Thorson, 1950). Such planktonic larvae may endow broad dispersal that should impede the divergence of populations and the formation of new species. Mayr (1954) proposed a strict allopatric solution to this problem. Surveying the geographic distributions of congeneric sea urchins, Mayr saw patterns that agreed “completely with terrestrial animals in which geographic speciation has been demonstrated”, that is, fully allopatric distributions of most congeneric species and sympatry only for taxa deemed long‐separated.

The geographic distributions of close relatives, however, do not all fit a model of strict allopatric speciation. Examining the geographic ranges of over 1,000 species of New World seaweeds, Pielou (1978) found the overlap between congeners to be greater than that of more distantly related taxa and noted an excess of congeners where the range of one species was nested within that of another. Subsequent molecular phylogenetic analyses found similar patterns (e.g. Hellberg, 1998; Hodge & Bellwood, 2016; Krug, 2011; Tavera et al., 2012; Taylor & Hellberg, 2005), again suggesting that the processes leading to species formation occurred within a relatively small region. Even some of Mayr's examples have eroded over time, with molecular analyses revealing some of the widespread “species” he examined to be composed of more geographically restricted cryptic taxa (e.g. *Echinometra*, Landry et al., 2003).

How then to explain the formation of new marine species with pelagic larval development without deep geographic divides? One way is to recognize that the geographic ranges of species are dynamic, such that the degree of overlap between sister species that we see today reveals little about their ranges during steps leading towards genetic isolation. Distant colonization events, geological change or the alternation of sea level or prevailing currents may produce transiently isolated populations. Divergence between these populations and their ancestral source could initiate reproductive isolation that was either continued or completed upon secondary contact. The genetic imprint of such diverge and reconnection may be well preserved in coastal species with limited dispersal (e.g. Marko, 1998). For species with pelagic larvae, such transient allopatry has been inferred by overlaying phylogenetic relationships with geographical history (Hellberg, 1998) or by polarizing phylogeographic patterns (Landry et al., 2003), but such suggestions are indirect at best.

More recently, interest has grown for an alternative scenario requiring no geographic isolation: ecological speciation, where divergent selection for performance in different habitats or on different resources produces reproductive isolation as a pleiotropic by‐product (Potkamp & Fransen, 2019). Hosts (Faucci et al., 2007; Hurt et al., 2013), prey (Moura et al., 2015) and exposure to waves and predators (Rolán‐Alvarez, 2007) have all been indicated as ecological drivers of such divergence, but the most common factor has been depth (Carlon & Budd, 2002; Gaither et al., 2018; Ingram, 2011; Prada & Hellberg, 2013). Indeed, depth has been noted as the most common ecological factor differentiating cryptic marine species (Knowlton, 1993).

Whether the course of speciation is completed in allopatry or sympatry, it must surmount a common barrier to differentiation: gene flow. Divergent selection can overwhelm the homogenizing effects of gene flow while allopatry escapes them altogether. Analyses that reveal the history of gene flow between populations (Sousa & Hey, 2013), then, should be able to distinguish between these alternatives. Simplistically, continuous isolation would favour an allopatric model while exchange throughout the divergence history would point to sympatric speciation. Actual results, however, do not map so easily to classic alternative models (see Bird et al., 2012). Still, emerging genomic analyses can expose the degree of isolation and exchange that have led to the emergence of genetically differentiated ecotypes (Foote et al., 2019). These analyses can also test for whether bouts of genetic exchange were symmetrical or directionally biased (Bertola et al., 2020) and whether exchange is uniform across the genome or there are “genomic islands” that experience less exchange (Rougemont et al., 2017). Finally, they can also be used to infer timing of demographic expansions (Prada et al., 2016; Takeuchi et al., 2020), that may be telling with regard to biotic and environmental changes that produce major changes in population size.

Here, we revisit a previous demographic analysis of two depth‐segregated, genetically isolated ecotypes of the Caribbean candelabrum coral *Eunicea flexuosa* (Lamouroux, 1821). The two ecotypes, Shallow and Deep, differ in several aspects of colony and spindle morphology (Prada et al., 2008). Both also show greater rates of survival in their native depth than when reciprocally transplanted (Prada & Hellberg, 2013). The ecotypes co‐occur at intermediate depths along with a small percentage of colonies of mixed heritage (Prada & Hellberg, 2014). Despite the small scale (<100 m) over which this habitat‐associated replacement occurs, each ecotype on its own appears panmictic at a Caribbean‐wide spatial scale (Prada & Hellberg, 2013). Analyses based upon one mitochondrial and three nuclear loci (Prada & Hellberg, 2013) weighed strongly against strict isolation and suggested recent exchange was primarily from the Shallow ecotype to the Deep, but could not distinguish among more subtle demographic models with varying degrees of isolation, exchange and population growth. Here, we employ >3,000 times more markers, along with more powerful demographic analyses that make use of the site frequency spectrum, to test over 100 alternative scenarios for divergence between the two ecotypes. We apply these same analyses to two other recent data sets reporting genetic differentiation between shallow and deep coral populations to test whether any trends in patterns of isolation, the timing and direction of genetic exchange, and population growth appear general. Together, our results suggest that even when gene flow connects deep and shallow populations over most of the course of their divergence, bouts of transient isolation still occur.

## 2 MATERIALS AND METHODS

### 2.1 Sampling, RNA extraction, library preparation and Illumina sequencing

To quantify genetic variation across depths, we sampled 59 colonies from both the Shallow (32) and the Deep ecotypes (27) from both their native depth and an intermediate depth in Parguera (Media Luna Reef), Puerto Rico. To confirm the Shallow and Deep ecotypes, we sequenced the 5′ end of the diagnostic mitochondrial *msh* gene using a previous protocol (Prada & Hellberg, 2013). In addition, we sampled one individual from the closely related species *Plexaura homomalla* (kukenthali form) (Bayer, 1961; Sánchez & Wirshing, 2005). We preserved all collected individuals in RNA later in at least 1:4 volume. We kept samples at 6°C in a cooler with ice for 12 hr, then changed the RNAlater and stored the samples at −80°C. Samples were shipped on dry ice to LSU and later to UC Davis, where RNA extractions were made.

We chose to use RNAseq to generate SNPs for our demographic analysis for two reasons. First, corals host many microbial symbionts, including single‐celled algae that can number >30,000 per cell. DNA from these symbionts must be filtered out or they can confound demographic analyses of their host. Algal symbionts can be screened if bleached or naturally symbiont‐free colonies are available to create a reference (e.g. Posbic Leydet et al., 2018), but that was not the case here. Genomic resources could be used to ensure SNPs are host specific, but these are lacking for taxa phylogenetically close to *E. flexuosa*. Transcriptomic data provided the best option. Second, these RNAseq data come from a larger reciprocal transplant experiment aimed at testing changes in gene expression.

To isolate high‐quality mRNA, we dissected ~25 mg of tissue and used the NEB^®^ magnetic isolation kit following the manufacturer's instructions. After a few cleaning steps, we produced enriched poly(A)^+^ RNA by hybridizing it with oligo (d)_25_ magnetic beads. We then coupled the extraction kit with the NEBNext^®^ RNA Library Preparation Kit following the manufacturer's recommendations (except we used ½ reactions) with a final PCR enrichment step. Libraries were examined for quality with a Bioanalyzer 2100 (Agilent Technologies) and quantified with a fluorescent plate reader (Sinergy HTX). To avoid lane effects, we barcoded all samples with unique codes, pooled them and sequenced across six HiSeq 2000 (100 PE) lanes. Sequencing was done at the Vincent J. Coates Genomics Sequencing Laboratory (GSL) at UC Berkeley.

### 2.2 RNAseq data sequencing, SNP calling and SNP filtering

We cleaned and trimmed raw reads as necessary (often six bases at the 5′ end) using Trimmomatic‐0.33 (Bolger et al., 2014). We reassessed trimmed reads with Fast‐QC 0.10.1 and checked for quality (http://www.bioinformatics.babraham.ac.uk/projects/fastqc/ ). Before assembling the transcriptome, we removed bacterial RNA and *Symbiodinium* contamination with Deconseq‐graph_0.1 (Li et al., 2010), using BWA 0.5.9‐r16 (Li & Durbin, 2009) to align each read. To clean reads, we generated a database from NCBI’s bacterial rRNA database coupled with the *S. minutum* genome (Shoguchi et al., 2013) and transcriptomes (http://medinalab.org/zoox/). We retained the unmatched reads for downstream analysis.

To generate an unfolded site frequency spectrum, we built a transcriptome for *P. homomalla*. We combined all cleaned unmatched reads from *P. homomalla* to assemble the transcriptome using Trinity 2.1.1 (Grabherr et al., 2011) with normalization and Trimmomatic flags. Mapping efficiency was almost identical (>85% after cleaning) for either the *E. flexuosa* Shallow or Deep types as sequence divergence between lineages is <1%. To avoid mapping reads against multi‐copy genes, we identified single copy sequences by running OrthoFinder (Emms & Kelly, 2015) and kept those for subsequent analysis.

After assembling our mapping transcriptome, we aligned all cleaned reads to it using the BWA mem algorithm version 0.7.12 (Li & Durbin, 2009) and then generated sorted bam files using SAMtools 1.9. To avoid biases from misalignments and mapping, we conservatively used the Genome Analysis Toolkit (GATK) Version 4.1.2.0 (McKenna et al., 2010) and followed their best practices (DePristo et al., 2011). The sorted BAM files generated from BWA were used to feed GATK. In GATK, we initially Add read groups, sort and create indexes using the feature AddOrReplaceReadGroups, then marked PCR duplicates with picard.jar. We then used a new GATK tool called SplitNCigarReads developed specially for RNAseq, which splits reads into exon segments (getting rid of Ns but maintaining grouping information) and hard‐clipping any sequences overhanging into the intronic regions.

To call SNPs, we used *Freebayes* v 1.2.0 (Garrison & Marth, 2012). VCF files were then filtered using vcftools v 0.1.17 (Danecek et al., 2011) and *vcflib* (Garrison & Marth, 2012). We eliminated under‐sequenced individuals with <50% of the SNPs and filtered SNPs within 30 bp of indels (using bcftools v 1.9‐174‐g4caf1fd). We retained only SNPs that were biallelic, had a minDP of 5, had a minQ of 30 and a max‐missing <0.9 per population. To avoid false positives, we retained SNPs in which each allele is present in at least 20% of the reads at that site (i.e. a 20%–80% balance between variants for each SNP). We controlled for Hardy–Weinberg disequilibrium within each population using a *p*‐value exclusion threshold of.001 and retained only one SNP per 1,000 bp (approximately one per scaffold), avoiding linked SNPs.

### 2.3 Demographic inference

To quantify variation in demographic history, we used ordinary differential equations to model the evolution of allele frequencies as implemented in *moments* (Jouganous et al., 2017). While many loci may be subject to subtle selective divergence between close taxa (Westram et al., 2018), models in *moments* can include parameters for genomic islands of divergence and estimate the proportion of SNPs within them, which should both be a less disruptive way of caging potential effects of selection than removing outlier loci before estimating the AFS and provide a picture of how the proportion of loci residing in genomic islands changes through time (Roux et al., 2013).

To transform the filtered VCF file to *moments* format, we used the *vcf2dadi.pl* script (available: https://groups.google.com/forum/#!searchin/dadi‐user/vcf2dadi.pl/dadi‐user/kvzhF4XSyng/idVM5lLUpt0J). Using the *P. homomalla's* transcriptome as an outgroup, we then generated the unfolded SFS using *moments* (Jouganous et al., 2017). To capture information from most SNPs and to compensate for missing data, we used the python script easySFS (https://github.com/isaacovercast/easySFS) and projected the SFS to 48 (Deep) and 58 (Shallow) alleles. To account for variability in the SFS estimation, we used a nonparametric bootstrapping by resampling the SNP file generated from *vcf2dadi.pl* five times using dadiBoot.pl (available: https://groups.google.com/forum/#!searchin/dadi‐user/vcf2dadi.pl/dadi‐user/kvzhF4XSyng/idVM5lLUpt0J).

We explicitly tested 107 demographic models (described at https://github.com/z0on/AFS‐analysis‐with‐moments/) in *moments*. Briefly, the models for two diverging populations can include up to three historical epochs, during which effective population size can vary from other epochs. Parameters for genetic exchange can also vary among epochs and may be symmetrical or asymmetrical. SNPs can be partitioned into two classes: those belonging to genomic islands that experience enhanced differentiation or limited exchange and the rest of the genome. Critically, multiple models not only allow for divergence with gene flow (parameters for genetic exchange in all epochs) and various forms of secondary contact (genetic exchange subsequent to an epoch of isolation) but can account for changes in population size in ancestral and post‐split populations. Simulations (Momigliano et al., 2020) suggest that failing to account for such past changes in population size can bias model choice towards secondary contact.

To rank the different models according to the log‐likelihoods and number of parameters, we estimated the evidence ratio from the best model following (Anderson, 2008). Specifically, for each model, we ran *moments* at least 10 times for each of the five bootstrap replicates and recorded the inferred model parameters with the lowest likelihood score across the 107 models. We then inferred the Akaike information criterion (AIC) scores, differences between each model against the best model, the relative likelihood of each model given the data, the model probability and the evidence ratio in favour of each model. We used the evidence ratio to rank each model and followed Anderson (2008) for model selection. Demographic curves are scaled by a per‐generation mutation rate of 1.02 × 10^–9^ (Prada, DeBiasse, et al., 2014; Prada et al., 2016; Voolstra et al., 2011). Assuming a conservative annual growth rate of 4 cm (Beiring & Lasker, 2000; Prada et al., 2008; Yoshioka & Yoshioka, 1991), the generation time for *E. flexuosa* is 5 years.

### 2.4 Patterns of shared ancestry and admixture

To understand the structure of our genomic data, we first transformed our VCF into a genlight object and assessed missingness and distribution of SNPs using the R package *Adeneget* (Jombart et al., 2010). We then inferred population genetic structure using principal components analysis of genotypes (PCA) and inferred genetic clusters (*k* = 2) in *adeneget* v. 2. 1.1 for a dataset of 15,640 SNPs. We plotted samples using PC1 and PC2. To better understand the structure of the populations across depth gradients, we performed a discriminant analysis of principal components (DAPC) in *Adegenet* v. 2.1.1, partitioning the variance into that between‐ and within‐groups to maximize discrimination. The number of clusters inferred was estimated by 1,000 iterations of the K‐means clustering between *K* = 1 and 3 after retaining all PCs, and selecting the optimal number of PCs by 1,000 replicates of the *a*‐score. We also inferred neighbour‐joining trees using the R package phangorn v2.5.5 (Schliep, 2010).

### 2.5 Additional datasets

We also explored two other systems on coral reefs for which (a) shallow and deep populations of what has been a single nominal species are genetically differentiated and (b) genomic data (>1,000 SNP loci) were available.

The first is the *Agaricia fragilis* system in Bermuda (Bongaerts et al., 2017). Their final VCF file had been filtered for minor allele frequencies, which could distort the SFS. We used their VCF file “afra_2f.vcf”, then applied filters as described for *E. flexuosa*. The data were collected from four locations across Bermuda. All samples were identified as shallow (S) or deep (D) except individuals AFMPX6982H and AFMPD6982H, as we were unable to tell their sampling location or whether it was a mismatched individual, respectively. Our final data set consisted of nextRAD‐derived 8,898 SNPs across 93 individuals (52 from deep and 41 from shallow).

The second data set is from *Pocillopora damicornis* collected from the shallow reef flat (F) and deeper reef slope (S) areas on Heron Island in the southern Great Barrier Reef (van Oppen et al., 2018). They genotyped 94 individuals (48 from the reef flat, 46 from the reef slope) for 2,091 SNPs derived from a RAD‐seq screen. We used the VCF file they had uploaded to (https://datadryad.org/stash/dataset/doi:10.5061/dryad.j09c05c).

## 3 RESULTS

**Figure 1:**
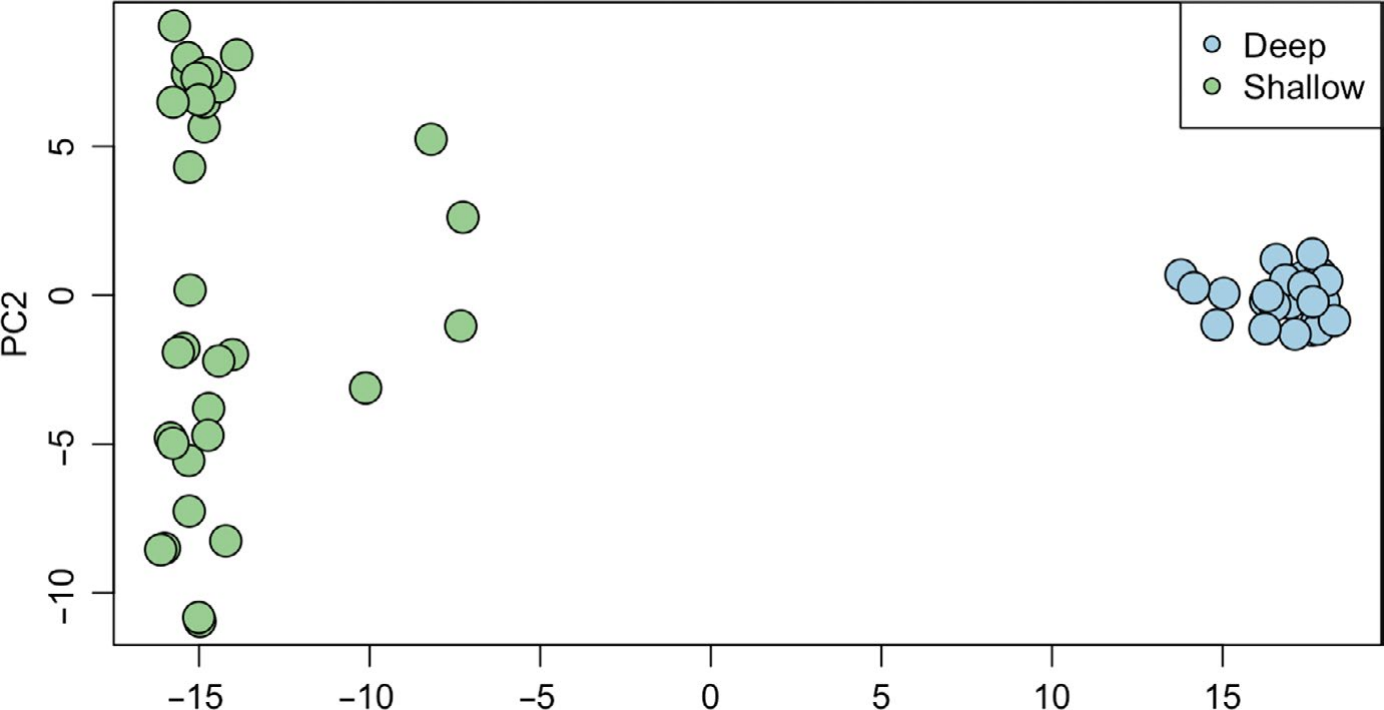
Principle Components Analysis for SNP data from Deep and Shallow ecotypes of *Eunicea flexuosa*

A total of 1.79 × 10^9^ 100‐bp PE raw reads were generated from the 59 individuals of *E. flexuosa* (Table S1). After trimming and cleaning, 3.30 × 10^8^ reads were aligned to the reference transcriptome of *P. homomalla*, resulting in the mapping of 1.65 × 10^8^ reads. Transcriptome‐wide nucleotide diversity was high: 0.115 for the Shallow ecotype and 0.113 for the Deep. After filtering out paralogues and bad quality SNPs, then retaining only one SNP per 1 Kb, our final data set consisted of 15,640 SNPs. PCA suggests that the two ecotypes of *E. flexuosa* are genetically distinct (Figure 1), with Deep type individuals being more tightly clustered than Shallow ones.

**Figure 2:**
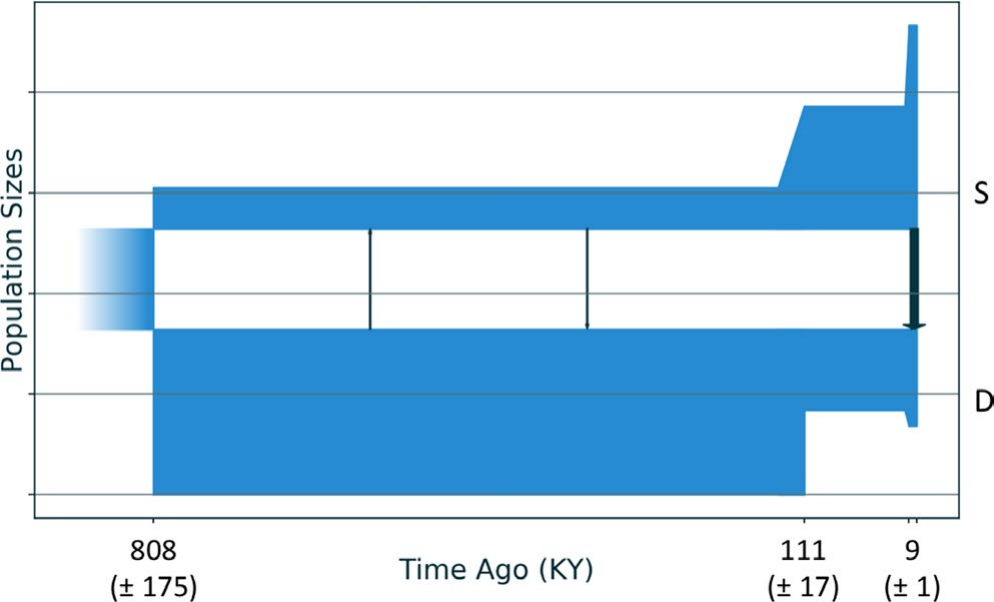
Best‐fit demographic model from *moments* analysis for the history of demography and genetic exchange between the Shallow (S) and Deep (D) ecotypes of *Eunicea flexuosa*. This best model (sc3imilm1) includes three epochs: an initial split, with asymmetric migration in the most recent epoch, no migration in the middle epoch, and low levels of symmetric migration in the earliest epoch. Horizontal lines are separated by 12,650 individuals. Standard deviations for date estimates are below in parentheses

**Figure 3:**
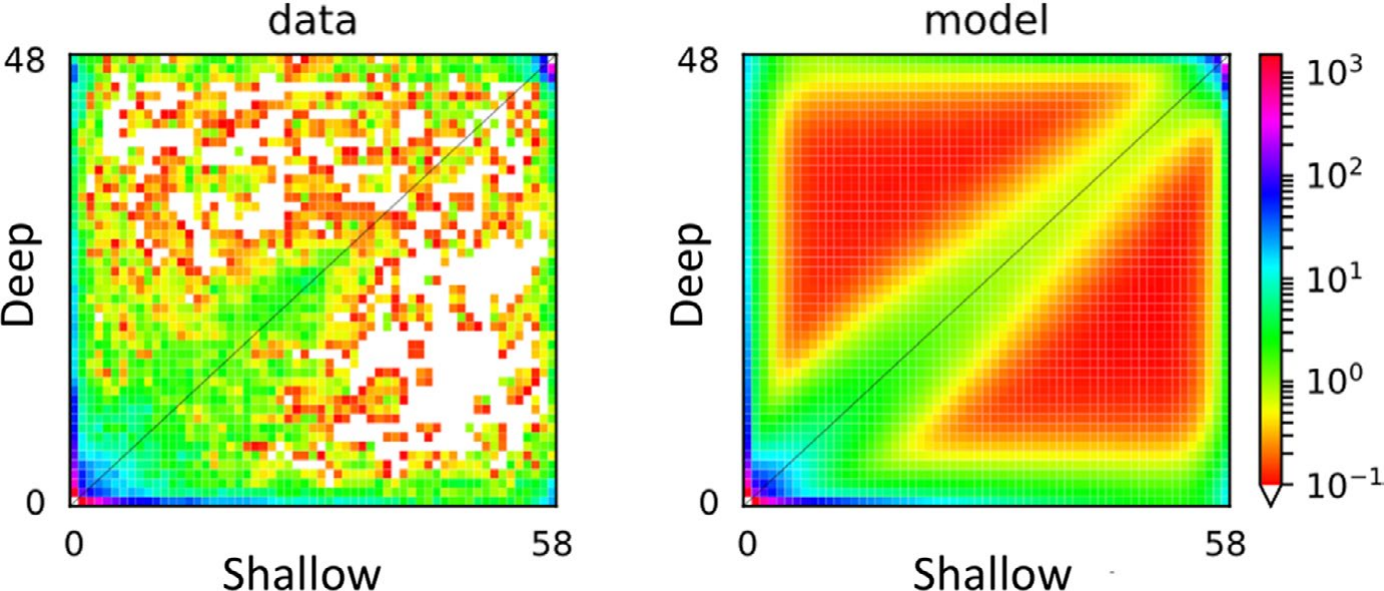
2D allele frequency spectra from *Eunicea flexuosa* for the data (left) and the model (right). The colour scale indicates how many SNPs occur for each combination of Deep (vertical axis) and Shallow (horizontal) ecotype alleles

The best‐fit demographic model for the recent history of the *E. flexuosa* pair (sc3ielsm1, Figure 2) was far better supported (evidence ratio > 10^40^) than any alternative (Table S2). The 2D allele frequency spectrum (Figure 3) showed a high density of SNPs with similar allele frequencies between Shallow and Deep populations (around the diagonal), but the presence of markers at the edges of the spectrum suggest that some regions of the genome display strong differences in allele frequencies between the ecotypes. The narrow range of the residuals between the model and the data (Figure S1) suggests strong power for the best model to predict the observed data.

This best‐fit model (Figure 2) included terms for three epochs in each population after an initial split. Divergence was initiated about 808 Kya, followed by 700 Kya of low but symmetrical genetic exchange. Genetic isolation marked a middle epoch that began 111 Kya, with the population size of the Deep form contracting as the Shallow expanded. The most recent epoch began 8.5 Kya, with asymmetrical gene flow from Shallow into Deep as both populations expanded. The model also included a term allowing differential levels of exchange between genomic islands and the rest of the genome. Islands of limited exchange held just 0.00122% of loci during the initial epoch of divergence with gene flow but grew to 24.4% during the most recent epoch. The second‐best supported model (sc3imlsm1) differed from the best only in that isolation occurred during the first historical epoch rather than the second one. The best model that included no epoch of isolation (iMi) was ranked 27th among all models tested.

**Figure 4:**
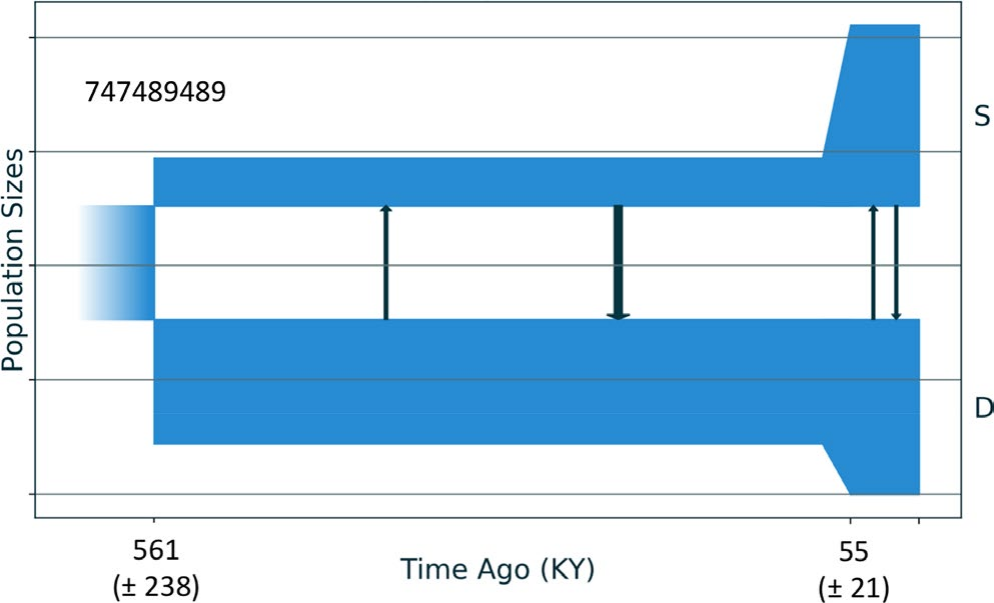
Best‐fit demographic model (sc2ielsm2) from *moments* analysis for the history of demography and genetic exchange between shallow (S) and deep (D) populations of *Agaricia fragilis* based on data from (Bongaerts et al., 2017). This best model included two epochs in each population, with asymmetric migration in the first and symmetric in the second. Both populations experienced expansions beginning 55 Kya. Horizontal lines are separated by 74,894 individuals

In the best‐fit demographic model for shallow and deep populations of *Agaricia fragilis* (sc2ielsm2), divergence with gene flow is estimated to have begun 561 Kya, leading into a 500+ Ky epoch of asymmetrical gene flow, primarily from Shallow to Deep (Figure 4 and Figure S2). Both populations experienced population expansions beginning 221 Kya. Gene flow during this more recent epoch has been symmetrical and at levels lower than before. The best‐fit model also included a term allowing differential levels of exchange between genomic islands of differentiation and the rest of the genome. This best model was 2.5 times more likely than the next‐best alternative (sc12il) and over five times better than the third best (sc12imlsm2) (Table S3). These next‐best alternatives differed from the best in that they included a predivergence episode of population growth. The probability weight for these three best‐fit models accounted for 85% (Table S3). The genetic distinctiveness of the two *A. fragilis* populations was not as great as for *E. flexuosa* (Figure S1) and may contribute to the inability to identify a single, clear‐cut best‐fit model.

**Figure 5:**
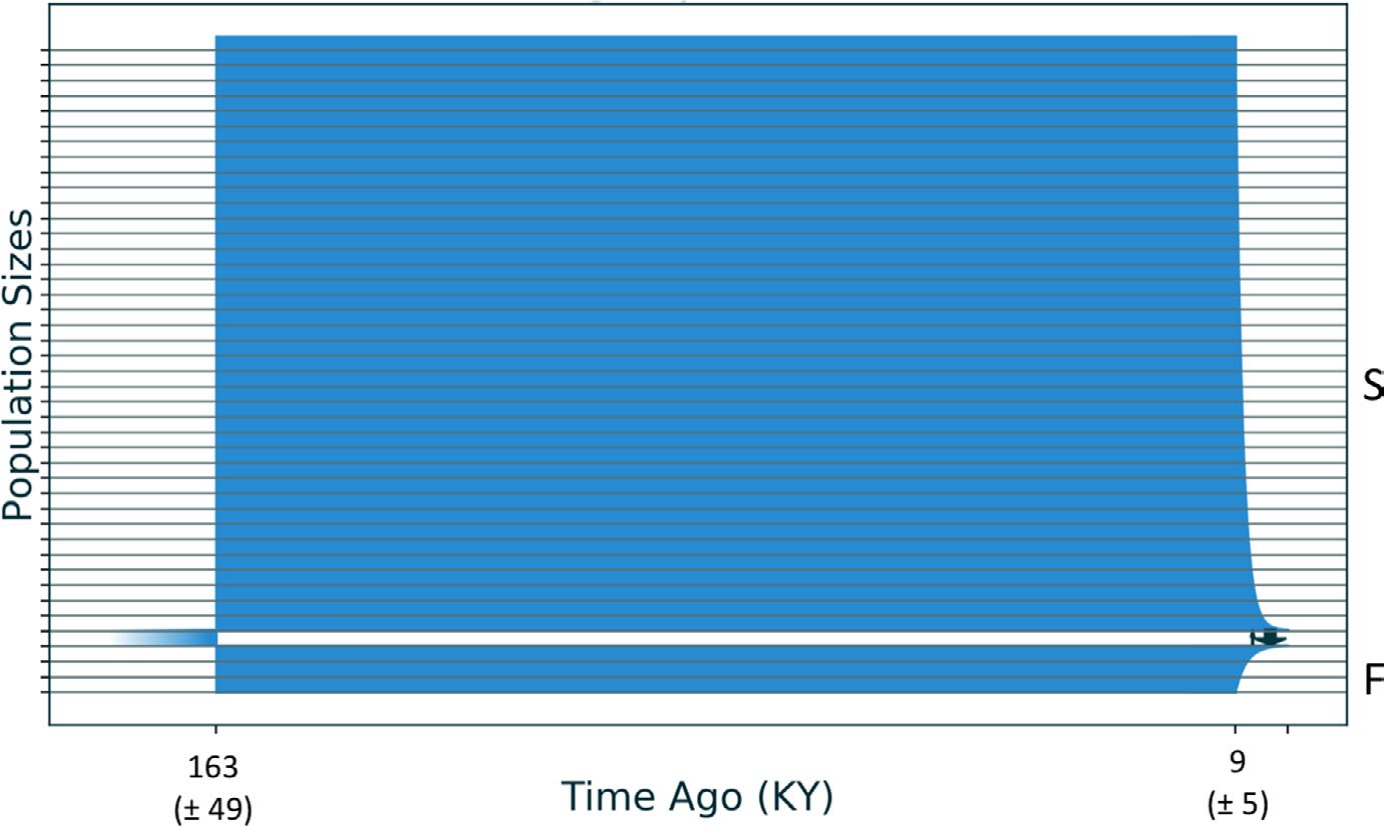
Best‐fit demographic model (IMisc) from *moments* analysis for the history of demography and genetic exchange between deep reef slope (S) and shallower reef flat (F) populations of *Pocillopora damicornis* based on data from (van Oppen et al., 2018). This best model included two epochs in each population, with symmetric migration in first epoch and asymmetric in second epoch. Horizontal lines are separated by 42,771 individuals

Several alternative models for the demographics of reef slope and reef flat populations of *Pocillopora damicornis* had similar levels of support (Table S4). The best‐fit model (IMisc) had a 40% probability. It suggested that divergence began in isolation 163 Kya (Figure 5), with population sizes remaining stable since then. Genetic exchange has occurred only recently, being higher in the deep‐to‐shallow direction. The two next‐best models, Sc2il and Sc2ilsm, suggest isolation with recent gene flow, but model Sc2i is different in that initial divergence occurred with gene flow. All models (except Sc2ilsm) favour a minor exchange in the direction from slope to flat. All four of these models are within a 10‐fold evidence ratio, so distinguishing between those with initial full isolation and isolation with gene flow is equivocal. As with *A. fragilis*, the slope and flat populations of *P. damicornis* were less genetically distinctive than in *E. flexuosa* (Figure S3) and this data set also contained the fewest SNPs. As for the other two species, all of the best‐fit models also included a term allowing differential levels of exchange between genomic islands of differentiation and the rest of the genome.

## 4 DISCUSSION

Previous work on *Eunicea flexuosa* revealed that it is composed of two genetically distinct populations, each adapted to the depth where it is most common (Prada & Hellberg, 2013; Prada et al., 2008). Demographic analysis (IMa) based on four genetic markers rejected a model of strict allopatric divergence between them. Based on these results, we suggested that this depth‐segregated pair may have diverged despite continuous gene flow via immigrant inviability, enhanced by the extended time habitat‐specific selection has to act between larval settlement and adulthood (Prada & Hellberg, 2013, 2014).

While this scenario is plausible, subsequently emerging data, analyses and insights call it into question. First, rejecting strict allopatry need not imply a model of divergence‐with‐(continuous)‐gene‐flow, as more complicated histories of transient genetic isolation may still have occurred (Yang et al., 2017). More generally, the inferred monophyly of ecologically distinct sympatric species does not imply that they evolved in situ, as gene flow following the critical events of speciation could also produce such a pattern (Foote et al., 2019).

Here, we have revisited the history of demography and genetic interchange between the Shallow and Deep ecotypes of *E. flexuosa* using an analysis (*moments*) that allows for more complex patterns and with over 3,000 times as many genetic markers as before. This large number of markers provides a robust estimate of the site frequency spectrum, the basis for demographic analyses (like *moments*) that perform best at recent time scales (Patton et al., 2019). The best‐fit model of historical demography and interchange between the Shallow and Deep ecotypes of *E. flexuosa* (Figure 3) began with a long period of divergence with gene flow, but also included an episode of isolation before more recent asymmetrical gene flow primarily from the Shallow ecotype to the Deep.

Few other marine species segregated by depth have had their histories of genetic exchange examined by genomic markers permitting a similar level of demographic detail. The best models inferred for two co‐occurring Caribbean sea anemones (*Bartholomea annulata* Clades 1 and 2) using *moments* all supported divergence‐with‐gene‐flow (Titus et al., 2019), as did fastsimcoal2, although support for the latter was less than three‐times better than a model with secondary contact. These two clades of anemones are not known to be depth‐segregated; however, the relative abundance of Clade 1 in the murky waters off Bocas del Toro, Panama, may suggest it generally inhabits deeper waters. The best models for divergence between reef flat and reef slope populations of *Pocillopora damicornis* (Figure 5) point to isolation followed by recent gene flow, although without strong enough support to draw firm conclusions. Discriminating between alternatives was likewise difficult for the *Agaricia fragilis* data (Figure 4). Modest divergence and relatively high levels of contemporary genetic exchange may leave the demographic history of divergence shrouded in such cases, especially for neutral loci (Bierne et al., 2013).

The best‐fit model for *E. flexuosa* also suggests a recent (9 Ky BP) population expansion for both ecotypes. Such a pattern is common to many Caribbean reef‐associated species (Prada et al., 2016), from crypto‐benthic fish (Eytan & Hellberg, 2010) to sea turtles (Reid et al., 2019), and is correlated with an increase in available shelf area tied to sea level rise that followed the last glacial maximum (Bellwood & Wainwright, 2002). Still, dates inferred from such analyses should be interpreted with caution. Estimating generation times for long‐lived clonal organisms like *E. flexuosa* is complicated by the interaction between age and size: larger, older genets may contribute disproportionately to population‐wide reproductive output. Selection can also influence molecular dating. RNAseq‐derived markers may be under negative (stabilizing) selection. If so, genetic diversity would be lower than for truly neutral markers (Charlesworth, 1996), biasing estimates of population size downward and those for divergence times to be older (although accelerated rates of lineage sorting in regions of reduced N_e_ would also tend to underestimate divergence times).

The symmetry of gene flow also varied over the course of divergence. While genetic exchange in the early steps towards speciation was inferred to be symmetric for *E. flexuosa* (Figures 2 and 3), recent post‐isolation exchange was asymmetrical. Similar patterns of recent asymmetric introgression have been reported for sea anemones (Titus et al., 2019), snapping shrimp (Hurt et al., 2013), oysters (Gagnaire et al., 2018) and flatfish (Souissi et al., 2018). Migration was also strongly asymmetric for sympatric ecomorphs of the European whitefish in aquatic settings (Rougeux et al., 2019). Such asymmetric genetic exchange may result from differences in population size between the two lineages and unaccounted‐for population expansions, but also from differences in the strength of reproductive barriers or from adaptive introgression. If barriers between the incipient species are largely complete, genes will slip through only when boosted by selection (e.g. Yang et al., 2018). Our best‐fit model for *E. flexuosa* shows proportion of loci residing in protected genomic islands increasing greatly between the first epoch of divergence with gene flow to the most recent, a pattern also seen and associated with asymmetric gene flow in European sea bass (Tine et al., 2014).

If asymmetric migration signals adaptive introgression, then the direction of gene flow suggests that recently adaptive genes have arisen in shallow habitats more often than deep: gene flow moves from shallow populations to deep ones in depth‐segregated corals, including *E. flexuosa* (Prada & Hellberg, 2013; Figure 2 above) and *Agaricia fragilis* (Bongaerts et al., 2017, Figure 4 above). A shallow‐to‐deep direction may indicate that genes favoured in warmer or more brightly lit surface waters have been spreading to deeper water. Notably, the Deep ecotype of *E. flexuosa* is more sensitive to warm water bleaching than the Shallow (Prada et al., 2010). As ocean warming continue, introgression from shallow populations may bring genotypes that allow deeper individuals to adapt to warmer oceans, although such introgression may also erode the distinctiveness of deep forms already suffering from warming conditions. Such a dilemma underlines the importance of recognizing cryptic species such as the *E. flexuosa* ecotypes, as ignoring differences in their susceptibility to climate change stressors can undervalue threats to local populations and ultimately to entire reef ecosystems (Fišer et al., 2018).

Depth provides a steep ecological gradient that can generate the strong differential selection required to drive divergence‐with‐gene‐flow. Despite this potential, our analysis of the Deep and Shallow ecotypes of *E. flexuosa* suggest that their divergence is still punctuated by an episode of transient isolation. Divergence‐with‐gene‐flow flanks both sides of this bout of isolation: before it is symmetrical, afterwards it is asymmetrical. Are there critical changes that cement the divergence of populations that occur only (or more readily) in isolation? As of yet, we do not know the genetic basis of the ecological and reproductive traits that segregate the two ecotypes. Outlier analyses for candidate genes associated with phenotypic divergence may help identify them. Given that information, and a genomic sampling of multiple individuals, new approaches could help to map and date selective sweeps driving divergence (e.g. Tournebize et al., 2019).

Finally, a full understanding of divergence with depth may require knowledge of the interactions between the coral host and its consortium of symbionts. In *E. flexuosa*, Shallow and Deep ecotypes harbour discrete populations of *Brevolium minutum* (Prada, McIlroy, et al., 2014) that may help confer their capacity to occupy different light niches. Studies of similar associations with bacteria are just beginning, but we know that some bacteria co‐diverge with their hosts (Pollock et al., 2018), that microbiomes vary across depth within species (Glasl et al., 2017) and that different lineages of the common coral symbiont *Endozoicomonas* rise and fall in correlation with environmental stressors in *E. flexuosa* (A. Reigel & M. E. Hellberg, unpub. data). Together, these suggest that multiple components of the coral holobiont play a role in adaptation with depth and potentially to giving rise to depth‐segregated reef species.

## CONFLICT OF INTEREST

All authors declare no conflict of interest.

## AUTHOR CONTRIBUTIONS

C. P. and M. E. H. designed the study and wrote the manuscript; C. P. performed the field and laboratory research and analysed the data.

### Peer Review

The peer review history for this article is available at https://publons.com/publon/10.1111/jeb.13731.

## Supporting information

Supplementary Material

## Data Availability

Reads have been deposited in the NCBI Short Read Archive under accession numbers SAMN16478884–SAMN16478945 (BioProject PRJNA669861).

## References

[jeb13731-bib-0001] Anderson, D. R. (2008). Model based inference in the life sciences. Springer.

[jeb13731-bib-0003] Bayer, F. M. (1961). The shallow‐water Octocorallia of the West Indian region. Studies on the Fauna of Curaçao and other. Caribbean Islands, 12, i–viii, 1–373.

[jeb13731-bib-0004] Beiring, E. A. , & Lasker, H. R. (2000). Egg production by colonies of a gorgonian coral. Marine Ecology Progress Series, 196, 169–177. 10.3354/meps196169

[jeb13731-bib-0005] Bellwood, D. R. , & Wainwright, P. C. (2002). The history and biogeography of fishes on coral reefs In P. F. Sale (Ed.), Coral reef fishes (pp. 5–32). Elsevier Science.

[jeb13731-bib-0006] Bertola, L. D. , Boehm, J. T. , Putman, N. F. , Xue, A. T. , Robinson, J. D. , Harris, S. , Baldwin, C. C. , Overcast, I. , & Hickerson, M. J. (2020). Asymmetrical gene flow in five co‐distributed syngnathids explained by ocean currents and rafting propensity. Proceedings of the Royal Society B: Biological Sciences, 287, 20200657 10.1098/rspb.2020.0657 PMC7282920 32370669

[jeb13731-bib-0007] Bierne, N. , Gagnaire, P.‐A. , & David, P. (2013). The geography of introgression in a patchy environment and the thorn in the side of ecological speciation. Current Zoology, 59, 72–86. 10.1093/czoolo/59.1.72

[jeb13731-bib-0008] Bird, C. E. , Fernandez‐Silva, I. , Skillings, D. J. , & Toonen, R. J. (2012). Sympatric speciation in the post "Modern Synthesis" era of evolutionary biology. Evolutionary Biology, 39, 158–180. 10.1007/s11692-012-9183-6

[jeb13731-bib-0009] Bolger, A. M. , Lohse, M. , & Usadel, B. (2014). Trimmomatic: A flexible trimmer for Illumina sequence data. Bioinformatics, 30, 2114–2120. 10.1093/bioinformatics/btu170 24695404 PMC4103590

[jeb13731-bib-0011] Bongaerts, P. , Riginos, C. , Brunner, R. , Englebert, N. , Smith, S. R. , & Hoegh‐Guldberg, O. (2017). Deep reefs are not universal refuges: Reseeding potential varies among coral species. Science Advances, 3, e1602373 10.1126/sciadv.1602373 28246645 PMC5310828

[jeb13731-bib-0012] Carlon, D. B. , & Budd, A. F. (2002). Incipient speciation across a depth gradient in a scleractinian coral? Evolution, 56, 2227–2242. 10.1111/j.0014-3820.2002.tb00147.x 12487353

[jeb13731-bib-0013] Charlesworth, B. (1996). Background selection and patterns of genetic diversity in *Drosophila melanogaster* . Genetical Research, 68, 131–149. 10.1017/S0016672300034029 8940902

[jeb13731-bib-0014] Danecek, P. , Auton, A. , Abecasis, G. , Albers, C. A. , Banks, E. , DePristo, M. A. , Handsaker, R. E. , Lunter, G. , Marth, G. T. , Sherry, S. T. , McVean, G. , & Durbin, R. (2011). The variant call format and VCFtools. Bioinformatics, 27, 2156–2158. 10.1093/bioinformatics/btr330 21653522 PMC3137218

[jeb13731-bib-0015] DePristo, M. A. , Banks, E. , Poplin, R. , Garimella, K. V. , Maguire, J. R. , Hartl, C. , Philippakis, A. A. , del Angel, G. , Rivas, M. A. , Hanna, M. , McKenna, A. , Fennell, T. J. , Kernytsky, A. M. , Sivachenko, A. Y. , Cibulskis, K. , Gabriel, S. B. , Altshuler, D. , & Daly, M. J. (2011). A framework for variation discovery and genotyping using next‐generation DNA sequencing data. Nature Genetics, 43, 491–498. 10.1038/ng.806 21478889 PMC3083463

[jeb13731-bib-0016] Emms, D. , & Kelly, S. (2015). OrthoFinder: Solving fundamental biases in whole genome comparisons dramatically improves orthogroup inference accuracy. Genome Biology, 16, 157 10.1186/s13059-015-0721-2 26243257 PMC4531804

[jeb13731-bib-0017] Eytan, R. I. , & Hellberg, M. E. (2010). Nuclear and mitochondrial sequence data reveal and conceal different demographic histories and population genetic processes in Caribbean reef fishes. Evolution, 64, 3380–3397. 10.1111/j.1558-5646.2010.01071.x 20584072

[jeb13731-bib-0018] Faucci, A. , Toonen, R. J. , & Hadfield, M. G. (2007). Host shift and speciation in a coral‐feeding nudibranch. Proceedings of the Royal Society London B: Biological Sciences, 274, 111–119. 10.1098/rspb.2006.3685 PMC1679885 17134995

[jeb13731-bib-0019] Fišer, C. , Robinson, C. T. , & Malard, F. (2018). Cryptic species as a window into the paradigm shift of the species concept. Molecular Ecology, 27, 613–635. 10.1111/mec.14486 29334414

[jeb13731-bib-0020] Foote, A. D. , Martin, M. D. , Louis, M. , Pacheco, G. , Robertson, K. M. , Sinding, M.‐H. , Amaral, A. R. , Baird, R. W. , Baker, C. S. , Ballance, L. , Barlow, J. , Brownlow, A. , Collins, T. , Constantine, R. , Dabin, W. , Dalla Rosa, L. , Davison, N. J. , Durban, J. W. , Esteban, R. , … Morin, P. A. (2019). Killer whale genomes reveal a complex history of recurrent admixture and vicariance. Molecular Ecology, 28, 3427–3444. 10.1111/mec.15099 31131963

[jeb13731-bib-0021] Gagnaire, P.‐A. , Lamy, J.‐B. , Cornette, F. , Heurtebise, S. , Dégremont, L. , Flahauw, E. , Boudry, P. , Bierne, N. , & Lapègue, S. (2018). Analysis of genome‐wide differentiation between native and introduced populations of the cupped oysters *Crassostrea gigas* and *Crassostrea angulata* . Genome Biology and Evolution, 10, 2518–2534. 10.1093/gbe/evy194 30184067 PMC6161763

[jeb13731-bib-0022] Gaither, M. R. , Gkafas, G. A. , de Jong, M. , Sarigol, F. , Neat, F. , Regnier, T. , Moore, D. , Grӧcke, D. R. , Hall, N. , Liu, X. , Kenny, J. , Lucaci, A. , Hughes, M. , Haldenby, S. , & Hoelzel, A. R. (2018). Genomics of habitat choice and adaptive evolution in a deep‐sea fish. Nature Ecology and Evolution, 2, 680–687. 10.1038/s41559-018-0482-x 29507380

[jeb13731-bib-0023] Garrison, E. & Marth, G. (2012). Haplotype‐based variant detection from short‐read sequencing. arXiv:1207.3907.

[jeb13731-bib-0024] Glasl, B. , Bongaerts, P. , Elisabeth, N. H. , Hoegh‐Goldberg, O. , Herndl, G. J. , & Frade, P. R. (2017). Microbiome variation in corals with distinct depth distribution ranges across a shallow–mesophotic gradient (15–85 m). Coral Reefs, 36, 447–452. 10.1007/s00338-016-1517-x 28579915 PMC5434129

[jeb13731-bib-0025] Grabherr, M. G. , Haas, B. J. , Yassour, M. , Levin, J. Z. , Thompson, D. A. , Amit, I. , Adiconis, X. , Fan, L. , Raychowdhury, R. , Zeng, Q. , Chen, Z. , Mauceli, E. , Hacohen, N. , Gnirke, A. , Rhind, N. , di Palma, F. , Birren, B. W. , Nusbaum, C. , Lindblad‐Toh, K. , … Regev, A. (2011). Full‐length transcriptome assembly from RNA‐Seq data without a reference genome. Nature Biotechnology, 29, 644–652. 10.1038/nbt.1883 PMC3571712 21572440

[jeb13731-bib-0026] Hellberg, M. E. (1998). Sympatric sea shells along the sea's shore: The geography of speciation in the marine gastropod *Tegula* . Evolution, 52, 1311–1324. 10.1111/j.1558-5646.1998.tb02013.x 28565375

[jeb13731-bib-0027] Hodge, J. R. , & Bellwood, D. R. (2016). The geography of speciation in coral reef fishes: The relative importance of biogeographical barriers in separating sister‐species. Journal of Biogeography, 43, 1324–1335. 10.1111/jbi.12729

[jeb13731-bib-0028] Hurt, C. , Silliman, K. , Anker, A. , & Knowlton, N. (2013). Ecological speciation in anemone‐associated snapping shrimps (Alpheus armatus species complex). Molecular Ecology, 22, 4532–4548. 10.1111/mec.12398 23859595

[jeb13731-bib-0029] Ingram, T. (2011). Speciation along a depth gradient in a marine adaptive radiation. Proceedings of the Royal Society B: Biological Sciences, 278, 613–618. 10.1098/rspb.2010.1127 PMC3025674 20810434

[jeb13731-bib-0030] Jombart, T. , Devillard, S. , & Balloux, F. (2010). Discriminant analysis of principal components: A new method for the analysis of genetically structured populations. BMC Genetics, 11, 94 10.1186/1471-2156-11-94 20950446 PMC2973851

[jeb13731-bib-0031] Jouganous, J. , Long, W. , Ragsdale, A. P. , & Gravel, S. (2017). Inferring the joint demographic history of multiple populations: Beyond the diffusion approximation. Genetics, 206, 1549–1567. 10.1534/genetics.117.200493 28495960 PMC5500150

[jeb13731-bib-0032] Knowlton, N. (1993). Sibling species in the sea. Annual Review of Ecology and Systematics, 24, 189–216. 10.1146/annurev.es.24.110193.001201

[jeb13731-bib-0033] Krug, P. J. (2011). Patterns of speciation in marine gastropods: A review of the phylogenetic evidence for localized radiations in the sea. American Malacological Bulletin, 29, 169–186. 10.4003/006.029.0210

[jeb13731-bib-0034] Landry, C. , Geyer, L. B. , Arakaki, Y. , Uehara, T. , & Palumbi, S. R. (2003). Recent speciation in the Indo‐West Pacific: Rapid evolution of gamete recognition and sperm morphology in cryptic species of sea urchin. Proceedings of the Royal Society of London B: Biological Sciences, 270, 1839–1847. 10.1098/rspb.2003.2395 PMC1691439 12964987

[jeb13731-bib-0035] Li, H. , & Durbin, R. (2009). Fast and accurate short read alignment with Burrows‐Wheeler transform. Bioinformatics, 25, 1754–1760. 10.1093/bioinformatics/btp324 19451168 PMC2705234

[jeb13731-bib-0036] Li, R. , Li, Y. , Zheng, H. , Luo, R. , Zhu, H. , Li, Q. , Qian, W. , Ren, Y. , Tian, G. , Li, J. , Zhou, G. , Zhu, X. , Wu, H. , Qin, J. , Jin, X. , Li, D. , Cao, H. , Hu, X. , Blanche, H. , … Wang, J. (2010). Building the sequence map of the human pan‐genome. Nature Biotechnology, 28, 57–63. 10.1038/nbt.1596 19997067

[jeb13731-bib-0037] Marko, P. B. (1998). Historical allopatry and the biogeography of speciation in the prosobranch snail genus *Nucella* . Evolution, 52, 757–774. 10.1111/j.1558-5646.1998.tb03700.x 28565241

[jeb13731-bib-0038] Mayr, E. (1954). Geographic speciation in tropical echinoids. Evolution, 8, 1–18. 10.1111/j.1558-5646.1954.tb00104.x

[jeb13731-bib-0040] McKenna, A. , Hanna, M. , Banks, E. , Sivachenko, A. , Cibulskis, K. , Kernytsky, A. , Garimella, K. , Altshuler, D. , Gabriel, S. , Daly, M. , & DePristo, M. A. (2010). The Genome Analysis Toolkit: A MapReduce framework for analyzing next‐generation DNA sequencing data. Genome Research, 20, 1297–1303. 10.1101/gr.107524.110 20644199 PMC2928508

[jeb13731-bib-0041] Momigliano, P. , Florin, A.‐B. , & Merila, J. (2020). Biases in demographic modeling affact our understanding of the process of speciation. bioRxiv, 10.1101/2020.06.03.128298 PMC8233503 33624816

[jeb13731-bib-0042] Moura, A. E. , Kenny, J. G. , Chaudhuri, R. R. , Hughes, M. A. , Reisinger, R. R. , de Bruyn, P. J. N. , Dahlheim, M. E. , Hall, N. , & Hoelzel, A. R. (2015). Phylogenomics of the killer whale indicates ecotype divergence in sympatry. Heredity, 114, 48–55. 10.1038/hdy.2014.67 25052415 PMC4815593

[jeb13731-bib-0043] Patton, A. H. , Margres, M. J. , Stahlke, A. R. , Hendricks, S. , Lewallen, K. , Hamede, R. K. , Ruiz‐Aravena, M. , Ryder, O. , McCallum, H. I. , Jones, M. E. , Hohenlohe, P. A. , & Storfer, A. (2019). Contemporary demographic reconstruction methods are robust to genome assembly quality: A case study in Tasmanian devils. Molecular Biology and Evoluition, 36, 2906–2921. 10.1093/molbev/msz191 PMC6878949 31424552

[jeb13731-bib-0044] Pielou, E. C. (1978). Latitudinal overlap of seaweed species: Evidence for quasi‐sympatric speciation. Journal of Biogeography, 5, 227–238. 10.2307/3038038

[jeb13731-bib-0045] Pollock, F. J. , McMinds, R. , Smith, S. , Bourne, D. G. , Willis, B. L. , Medina, M. , Thurber, R. V. , & Zaneveld, J. R. (2018). Coral‐associated bacteria demonstrate phylosymbiosis and co‐phylogeny. Nature Communications, 9, 4921 10.1038/s41467-018-07275-x PMC6250698 30467310

[jeb13731-bib-0046] Posbic Leydet, K. , Grupstra, C. G. B. , Coma, R. , Ribes, M. , & Hellberg, M. E. (2018). Host‐targeted RADSeq reveals genetic changes in the coral *Oculina patagonica* associated with range expansion along the Spanish Mediterranean coast. Molecular Ecology, 27, 2529–2543. 10.1111/mec.14702 29693297

[jeb13731-bib-0047] Potkamp, G. , & Fransen, C. H. J. M. (2019). Speciation with gene flow in marine systems. Contributions to Zoology, 88, 133–172. 10.1163/18759866-20191344

[jeb13731-bib-0048] Prada, C. , DeBiasse, M. B. , Neigel, J. E. , Yednock, B. , Stake, J. L. , Forsman, Z. H. , Baums, I. B. , & Hellberg, M. E. (2014). Genetic species delineation among branching Caribbean *Porites* corals. Coral Reefs, 33, 1019–1030. 10.1007/s00338-014-1179-5

[jeb13731-bib-0049] Prada, C. , Hanna, B. , Budd, A. F. , Woodley, C. M. , Schmutz, J. , Grimwood, J. , Iglesias‐Prieto, R. , Pandolfi, J. M. , Levitan, D. , Johnson, K. G. , Knowlton, N. , Kitano, H. , DeGiorgio, M. , & Medina, M. (2016). Empty niches after extinctions increase population sizes of modern corals. Current Biology, 26, 3190–3194. 10.1016/j.cub.2016.09.039 27866895

[jeb13731-bib-0050] Prada, C. , & Hellberg, M. E. (2013). Long prereproductive selection and divergence by depth in a Caribbean candelabrum coral. Proceedings of the National Academy of Sciences of the United States of America, 110, 3961–3966. 10.1073/pnas.1208931110 23359716 PMC3593850

[jeb13731-bib-0051] Prada, C. , & Hellberg, M. E. (2014). Strong natural selection on juveniles maintains a narrow adult hybrid zone in a broadcast spawner. The American Naturalist, 184, 702–713. 10.1086/678403 25438171

[jeb13731-bib-0052] Prada, C. , McIlroy, S. E. , Beltrán, D. M. , Valint, D. J. , Ford, S. A. , Hellberg, M. E. , & Coffroth, M. A. (2014). Cryptic diversity hides host and habitat specialization in a gorgonian‐algal symbiosis. Molecular Ecology, 23, 3330–3340. 10.1111/mec.12808 24863571

[jeb13731-bib-0053] Prada, C. , Schizas, N. V. , & Yoshioka, P. M. (2008). Phenotypic plasticity or speciation? A case from a clonal marine organism. BMC Evolutionary Biology, 8, 47 10.1186/1471-2148-8-47 18271961 PMC2275222

[jeb13731-bib-0054] Prada, C. , Weil, E. , & Yoshioka, P. M. (2010). Octocoral bleaching during unusual thermal stress. Coral Reefs, 29, 41–45. 10.1007/s00338-009-0547-z

[jeb13731-bib-0055] Reid, B. N. , Naro‐Maciel, E. , Torres Hahn, A. , FitzSimmons, N. N. , & Gehara, M. (2019). Geography best explains global patterns of genetic diversity and postglacial co‐expansion in marine turtles. Molecular Ecology, 28, 3358–3370. 10.1111/mec.15165 31264298

[jeb13731-bib-0056] Rolán‐Alvarez, E. (2007). Sympatric speciation as a by‐product of ecological adaptation in the Galician *Littorina saxatilis* hybrid zone. Journal of Molluscan Studies, 73, 1–10. 10.1093/mollus/eyl023

[jeb13731-bib-0057] Rougemont, Q. , Gagnaire, P.‐A. , Perrier, C. , Genthon, C. , Besnard, A.‐L. , Launey, S. , & Evanno, G. (2017). Inferring the demographic history underlying parallel genomic divergence among pairs of parasitic and nonparasitic lamprey ecotypes. Molecular Ecology, 26, 142–162. 10.1111/mec.13664 27105132

[jeb13731-bib-0058] Rougeux, C. , Gagnaire, P.‐A. , & Bernatchez, L. (2019). Model‐based demographic inference of introgression history in European whitefish species pairs. Journal of Evolutionary Biology, 32, 806–817. 10.1111/jeb.13482 31038776

[jeb13731-bib-0059] Roux, C. , Tsagkogeorga, G. , Bierne, N. , & Galtier, N. (2013). Crossing the species barrier: Genomic hotspots of introgression between two highly divergent Ciona intestinalis species. Molecular Biology and Evolution, 30, 1574–1587. 10.1093/molbev/mst066 23564941

[jeb13731-bib-0060] Sánchez, J. A. , & Wirshing, H. H. (2005). A field key to the identification of tropical Western Atlantic zooxanthellate octocorals (Octocorallia: Cnidaria). Caribbean Journal of Science, 41, 508–522.

[jeb13731-bib-0061] Schliep, K. P. (2010). phangorn: Phylogenetic analysis in R. Bioinformatics, 27, 592–593. 10.1093/bioinformatics/btq706 21169378 PMC3035803

[jeb13731-bib-0062] Shoguchi, E. , Shinzato, C. , Kawashima, T. , Gyoja, F. , Mungpakdee, S. , Koyanagi, R. , Takeuchi, T. , Hisata, K. , Tanaka, M. , Fujiwara, M. , Hamada, M. , Seidi, A. , Fujie, M. , Usami, T. , Goto, H. , Yamasaki, S. , Arakaki, N. , Suzuki, Y. , Sugano, S. , … Satoh, N. (2013). Draft assembly of the *symbiodinium minutum* nuclear genome reveals dinoflagellate gene structure. Current Biology, 23, 1399–1408. 10.1016/j.cub.2013.05.062 23850284

[jeb13731-bib-0063] Souissi, A. , Bonhomme, F. , Manchado, M. , Bahri‐Sfar, L. , & Gagnaire, P. A. (2018). Genomic and geographic footprints of differential introgression between two divergent fish species (*Solea* spp.). Heredity, 121, 579–593. 10.1038/s41437-018-0079-9 29713088 PMC6221876

[jeb13731-bib-0064] Sousa, V. , & Hey, J. (2013). Understanding the origin of species with genome‐scale data: Modeling gene flow. Nature Reviews Genetics, 14, 404–414. 10.1038/nrg3446 PMC5568773 23657479

[jeb13731-bib-0065] Takeuchi, T. , Masaoka, T. , Aoki, H. , Koyanagi, R. , Fujie, M. , & Satoh, N. (2020). Divergent northern and southern populations of the pearl oyster in the western Pacific revealed with genomic SNPs. Evolutionary Applications, 13, 837–853. 32211071 10.1111/eva.12905 PMC7086055

[jeb13731-bib-0066] Tavera, J. , P, A. , Balart, E. F. , & Bernardi, G. (2012). Molecular phylogeny of grunts (Teleostei, Haemulidae), with an emphasis on the ecology, evolution, and speciation history of New World species. BMC Evolutionary Biology, 12, 57 10.1186/1471-2148-12-57 22537107 PMC3472276

[jeb13731-bib-0067] Taylor, M. S. , & Hellberg, M. E. (2005). Marine radiations at small geographic scales: Speciation in neotropical reef gobies (*Elacatinus*). Evolution, 59, 374–385. 10.1554/04-590 15807422

[jeb13731-bib-0068] Thorson, G. (1950). Reproductive and larval ecology of marine bottom invertebrates. Biological Reviews, 25, 1–45. 10.1111/j.1469-185X.1950.tb00585.x 24537188

[jeb13731-bib-0069] Tine, M. , Kuhl, H. , Gagnaire, P.‐A. , Louro, B. , Desmarais, E. , Martins, R. S. T. , Hecht, J. , Knaust, F. , Belkhir, K. , Klages, S. , Dieterich, R. , Stueber, K. , Piferrer, F. , Guinand, B. , Bierne, N. , Volckaert, F. A. M. , Bargelloni, L. , Power, D. M. , Bonhomme, F. , … Reinhardt, R. (2014). European sea bass genome and its variation provide insights into adaptation to euryhalinity and speciation. Nature Communications, 5, 5770 10.1038/ncomms6770 PMC4284805 25534655

[jeb13731-bib-0070] Titus, B. M. , Blischak, P. D. , & Daly, M. (2019). Genomic signatures of sympatric speciation with historical and contemporary gene flow in a tropical anthozoan (Hexacorallia: Actinaria). Molecular Ecology, 28, 3572–3586. 10.1111/mec.15157 31233641

[jeb13731-bib-0071] Tournebize, R. , Poncet, V. , Jakobsson, M. , Vigouroux, Y. , & Manel, S. (2019). McSwan: A joint frequency spectrum method to detect and date selective sweeps across multiple population genomes. Molecular Ecology Resources, 19, 283–295. 10.1111/1755-0998.12957 30358170

[jeb13731-bib-0072] van Oppen, M. J. H. , Bongaerts, P. , Frade, P. , Peplow, L. M. , Boyd, S. E. , Nim, H. T. , & Bay, L. K. (2018). Adaptation to reef habitats through selection on the coral animal and its associated microbiome. Molecular Ecology, 27, 2956–2971. 10.1111/mec.14763 29900626

[jeb13731-bib-0073] Voolstra, C. R. , Sunagawa, S. , Matz, M. V. , Bayer, T. , Aranda, M. , Buschiazzo, E. , DeSalvo, M. K. , Lindquist, E. , Szmant, A. M. , Coffroth, M. A. , & Medina, M. (2011). Rapid evolution of coral proteins responsible for interaction with the environment. PLoS One, 6, e20392 10.1371/journal.pone.0020392 21633702 PMC3102110

[jeb13731-bib-0074] Westram, A. M. , Rafajlović, M. , Chaube, P. , Faria, R. , Larsson, T. , Panova, M. , Ravinet, M. , Blomberg, A. , Mehlig, B. , Johannesson, K. , & Butlin, R. (2018). Clines on the seashore: The genomic architecture underlying rapid divergence in the face of gene flow. Evolution Letters, 2, 297–309. 10.1002/evl3.74 30283683 PMC6121805

[jeb13731-bib-0075] Yang, M. , He, Z. , Shi, S. , & Wu, C.‐I. (2017). Can genomic data alone tell us whether speciation happened with gene flow? Molecular Ecology, 26, 2845–2849. 10.1111/mec.14117 28345182

[jeb13731-bib-0076] Yang, W. , While, G. M. , Laakkonen, H. , Sacchi, R. , Zuffi, M. A. , Scali, S. , Salvi, D. , & Uller, T. (2018). Genomic evidence for asymmetric introgression by sexual selection in gthe common wall lizard. Molecular Ecology, 27, 4213–4224. 10.1111/mec.14861 30192998

[jeb13731-bib-0078] Yoshioka, P. M. , & Yoshioka, B. B. (1991). A comparison of the survivorship and growth of shallow‐water gorgonian species of Puerto Rico. Marine Ecology Progress Series, 69, 253–260. 10.3354/meps069253

