# Supplementary material for: Speciation‐by‐depth on coral reefs: Sympatric divergence with gene flow or cryptic transient isolation?
Source: J Evol Biol. 2020 Nov 20;34(1):128–37. doi: 10.1111/jeb.13731 (PMC7894305; doi:10.1111/jeb.13731)
Supplement: Supplementary file 1 — Supplementary Material [file JEB-34-128-s001.docx]

**Table S1** - Tallies of RNA-seq reads for different individuals of *Eunicea flexuosa* used in this stud with each ecotype identified and the original depth at which it was found.

| **Code** | **Ecotype (depth in meters**) | **Raw total data** | **After filtering** | **Mapped** |
| --- | --- | --- | --- | --- |
| 55_11 | Deep (15) | 38,459,742 | 7,532,850.00 | 7,116,380.00 |
| 55_13 | Shallow (15) | 23,939,792 | 7,018,831.00 | 3,967,646.00 |
| 55_14 | Deep (15) | 26,431,536 | 5,216,367.00 | 4,538,902.00 |
| 55_15 | Deep (15) | 27,856,678 | 5,564,150.00 | 3,613,924.00 |
| 55_16 | Shallow (15) | 24,080,204 | 6,187,669.00 | 4,217,668.00 |
| 55_17 | Shallow (15) | 28,699,738 | 5,434,544.00 | 3,795,398.00 |
| 55_18 | Deep (15) | 26,595,110 | 5,375,786.00 | 5,012,584.00 |
| 55_19 | Shallow (15) | 7,941,956 | 4,662,858.00 | 5,438,070.00 |
| 55_20 | Deep (15) | 18,293,698 | 4,509,169.00 | 7,029,436.00 |
| 55_21 | Deep (15) | 38,275,430 | 5,418,739.00 | 4,641,688.00 |
| 55_24 | Shallow (5) | 27,368,362 | 5,826,413.00 | 2,473,376.00 |
| 55_25 | Shallow (5) | 37,495,736 | 13,037,753.00 | 6,595,210.00 |
| 55_31 | Deep (15) | 48,813,228 | 6,268,197.00 | 2,599,632.00 |
| D01 | Shallow (5) | 25,358,750 | 4,318,850.00 | 2,013,957.00 |
| D03 | Shallow (5) | 26,060,180 | 6,316,621.00 | 2,645,742.00 |
| D08 | Shallow (5) | 24,198,042 | 4,469,349.00 | 2,314,595.00 |
| D13 | Shallow (5) | 34,814,734 | 5,918,011.00 | 2,345,878.00 |
| D14 | Shallow (5) | 22,065,864 | 3,882,393.00 | 1,189,272.00 |
| D18 | Shallow (5) | 29,590,764 | 6,378,736.00 | 2,349,955.00 |
| D19 | Shallow (5) | 20,988,590 | 2,135,277.00 | 799,498.00 |
| D20 | Deep (22) | 46,533,214 | 12,899,552.00 | 4,899,585.00 |
| D24 | Shallow (5) | 31,033,002 | 3,570,989.00 | 1,569,249.00 |
| D29 | Shallow (5) | 26,086,614 | 4,907,264.00 | 1,666,717.00 |
| D52 | Deep (22) | 23,671,628 | 2,917,435.00 | 1,282,060.00 |
| D56 | Deep (22) | 19,090,598 | 3,376,347.00 | 1,637,583.00 |
| D58 | Deep (22) | 33,873,444 | 8,147,016.00 | 4,142,382.00 |
| D60 | Deep (22) | 34,486,218 | 7,532,850.00 | 3,249,025.00 |
| D62 | Shallow (22) | 30,729,122 | 7,018,831.00 | 2,832,125.00 |
| D64 | Deep (22) | 28,139,502 | 5,216,367.00 | 2,017,930.00 |
| D68 | Deep (22) | 29,116,680 | 5,564,150.00 | 2,570,719.00 |
| D70 | Deep (22) | 20,904,802 | 6,187,669.00 | 2,744,093.00 |
| D74 | Deep (22) | 38,581,076 | 5,434,544.00 | 2,322,424.00 |
| D78 | Deep (22) | 24,353,638 | 5,375,786.00 | 2,372,950.00 |
| D80 | Shallow (22) | 23,675,536 | 4,662,858.00 | 1,716,632.00 |
| D87 | Deep (22) | 25,696,198 | 4,509,169.00 | 2,497,238.00 |
| S03 | Shallow (5) | 31,726,854 | 5,418,739.00 | 2,383,319.00 |
| S07 | Shallow (5) | 26,590,060 | 5,826,413.00 | 1,509,872.00 |
| S12 | Shallow (5) | 49,228,046 | 13,037,753.00 | 6,037,915.00 |
| S13 | Shallow (5) | 30,140,900 | 6,268,197.00 | 2,675,093.00 |
| S14 | Shallow (5) | 5,688,892 | 1,269,157.00 | 503,235.00 |
| S19 | Shallow (5) | 32,939,190 | 5,439,363.00 | 1,889,916.00 |
| S20 | Shallow (5) | 34,586,654 | 6,649,469.00 | 3,542,574.00 |
| S21 | Shallow (5) | 4,770,464 | 1,013,222.00 | 315,241.00 |
| S22 | Shallow (5) | 26,241,048 | 5,571,818.00 | 1,807,202.00 |
| S25 | Shallow (5) | 27,716,542 | 6,259,342.00 | 2,796,174.00 |
| S35 | Shallow (5) | 18,999,864 | 4,150,286.00 | 1,714,676.00 |
| S40 | Shallow (5) | 20,126,794 | 2,370,447.00 | 1,099,020.00 |
| S52 | Deep (22) | 22,395,752 | 3,288,007.00 | 1,390,195.00 |
| S53 | Deep (22) | 24,316,126 | 4,152,245.00 | 1,427,897.00 |
| S56 | Deep (22) | 24,860,050 | 3,853,087.00 | 1,772,207.00 |
| S62 | Deep (22) | 20,964,106 | 3,336,341.00 | 1,587,027.00 |
| S65 | Deep (22) | 23,349,240 | 3,615,461.00 | 1,509,955.00 |
| S71 | Deep (22) | 25,393,390 | 4,422,495.00 | 2,185,785.00 |
| S75 | Deep (22) | 38,415,312 | 8,388,960.00 | 2,479,051.00 |
| S80 | Shallow (22) | 15,749,586 | 3,629,629.00 | 1,243,824.00 |
| S86 | Deep (22) | 23,299,928 | 3,989,401.00 | 1,904,646.00 |
| S87 | Shallow (22) | 31,460,348 | 6,586,313.00 | 2,203,037.00 |
| S89 | Deep (22) | 65,883,374 | 10,470,786.00 | 5,359,214.00 |
| S90 | Shallow (22) | 40,465,714 | 8,658,888.00 | 3,243,808.00 |
|  |  |  |  |  |
| **Total** |  | **1.79E+09** | **3.30E+08** | **1.65E+08** |

**Table S2** - Best-fit models of historical demography and genetic exchange for *Eunicea flexuosa*. The best-fit model fits the data over 10^40^-times better than the next best model. Model scores are shown down to the best model without any period of genetic isolation (iMi).

**model* # para**^†^**log L**^‡^ **AIC**^§^ **wts**^¶^ **evidence ratio**

sc3ielsm1 17 -1915.85 3865.70 1 1

sc3imlsm1 17 -2009.49 4052.97 2.16E-41 4.63E+40

sc2i 16 -2020.46 4072.91 1.01E-45 9.87E+44

IMisc2 14 -2023.20 4074.40 4.81E-46 2.08E+45

IMiscsmB 11 -2028.61 4079.22 4.32E-47 2.31E+46

sc3il 15 -2038.24 4106.48 5.21E-53 1.92E+52

sc2ielsm2 14 -2040.90 4109.80 9.87E-54 1.01E+53

sc12imlsm1 16 -2041.01 4114.02 1.20E-54 8.36E+53

IMisc2sm 12 -2056.20 4136.41 1.65E-59 6.06E+58

sc3ielsm 13 -2056.41 4138.81 4.96E-60 2.02E+59

IMiscsm 10 -2062.06 4144.12 3.49E-61 2.86E+60

sc3ieml 15 -2057.60 4145.19 2.04E-61 4.89E+60

sc3imlsm 13 -2060.04 4146.08 1.31E-61 7.62E+60

sc3ielsm2 17 -2057.93 4149.86 1.98E-62 5.05E+61

sc3ilsm 13 -2062.14 4150.29 1.60E-62 6.26E+61

sc3iel 15 -2060.23 4150.46 1.47E-62 6.82E+61

sc12ilsm 12 -2068.03 4160.06 1.21E-64 8.28E+63

sc2ilsm 10 -2073.25 4166.49 4.84E-66 2.07E+65

sc3iemlsm 13 -2077.76 4181.53 2.63E-69 3.81E+68

sc3iml 15 -2083.86 4197.72 8.01E-73 1.25E+72

sc12iml 14 -2085.62 4199.25 3.74E-73 2.68E+72

sc2ielsm 10 -2092.93 4205.86 1.37E-74 7.31E+73

sc12imlsm 12 -2093.91 4211.82 6.95E-76 1.44E+75

sc3imlsm2 17 -2088.93 4211.85 6.84E-76 1.46E+75

IMisc 12 -2096.38 4216.76 5.89E-77 1.70E+76

IMi 11 -2099.92 4221.84 4.63E-78 2.16E+77

*A key to the meanings of the different model abbreviations can be found at: <https://github.com/z0on/AFS-analysis-with-moments/blob/master/multimodel_inference/moments_multimodels.xlsx>

† - #para - number of parameters in the *moments* demographic model

‡ - log L - log likelihood of the demographic model

§ - AIC - Akaike information criterion

¶ - wts - model weight against all models

**Table S3** - Best-fit models of historical demography and genetic exchange for *Agaricia fragilis*. See Table S2 for abbreviations. Model scores are shown down to model with a probability >0.001.

**model npara log L AIC wts evi_ratio**

sc2ielsm2 13 -1488.79 3003.57 0.54 1

sc12il 13 -1489.73 3005.46 0.21 2.57

sc12imlsm2 15 -1488.45 3006.91 0.10 5.30

sc3il 14 -1490.08 3008.17 0.05 9.95

sc3ielsm1 16 -1488.60 3009.21 0.03 16.7

sc12imlsm1 15 -1489.90 3009.81 0.02 22.6

sc12ilsm 11 -1494.17 3010.34 0.02 29.4

sc3imlsm1 16 -1490.51 3013.02 0.00 113

IMisc2 13 -1493.83 3013.66 0.00 155

sc3imlsm2 16 -1490.86 3013.72 0.00 160

sc2il 11 -1495.89 3013.79 0.00 165

IMisc 11 -1496.73 3015.46 0.00 381

sc3ielsm 12 -1496.47 3016.94 0.00 799**Table S4** - Best-fit models of historical demography and genetic exchange for *Pocillopora damicornis*. See Table S2 for abbreviations. Model scores are shown down to model with a probability >0.001.

| **model** | **npara** | **log L** | | **AIC** | **wts** | **evi_ratio** |
| --- | --- | --- | --- | --- | --- | --- |
| IMisc | 11 | -811.52 | 1645.049 | | 0.400 | 1 |
| sc2il | 11 | -812.21 | 1646.413 | | 0.202 | 1.98 |
| sc2ilsm | 9 | -814.24 | 1646.484 | | 0.195 | 2.05 |
| sc2i | 15 | -809.12 | 1648.235 | | 0.081 | 4.92 |
| IMiscsm | 9 | -815.62 | 1649.232 | | 0.049 | 8.10 |
| sc2ielsm1 | 13 | -812.27 | 1650.534 | | 0.026 | 15.5 |
| IMsc | 8 | -818.79 | 1653.579 | | 0.0056 | 71.1 |
| IMiscsmB | 10 | -816.87 | 1653.739 | | 0.0052 | 77.1 |
| IMsc | 8 | -818.92 | 1653.845 | | 0.0049 | 81.3 |
| IMsc | 8 | -819.05 | 1654.105 | | 0.0043 | 92.5 |
| sc2ielsm2 | 13 | -814.15 | 1654.306 | | 0.0039 | 102 |
| sc3imlsm2 | 16 | -811.18 | 1654.357 | | 0.0038 | 105 |
| sc2ilsm | 9 | -818.31 | 1654.625 | | 0.0033 | 120 |
| sc2i | 15 | -812.35 | 1654.705 | | 0.0032 | 125 |
| sc3imlsm2 | 16 | -811.70 | 1655.395 | | 0.0023 | 176 |
| IMisc | 11 | -816.99 | 1655.985 | | 0.0017 | 237 |
| sc11 | 7 | -821.16 | 1656.311 | | 0.0014 | 279 |
| sc11 | 7 | -821.29 | 1656.585 | | 0.0013 | 320 |
| IMiscsm | 9 | -819.36 | 1656.718 | | 0.0012 | 342 |


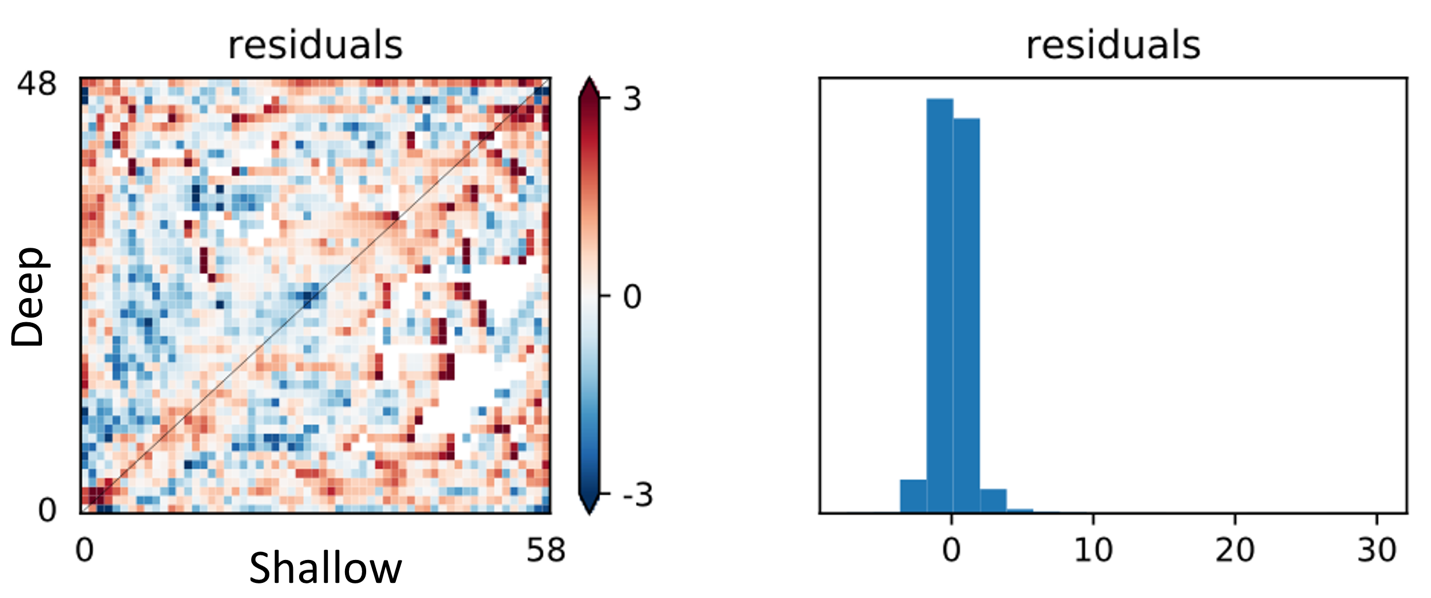


**Figure S1.** The 2D frequency spectrum of residuals (left) and frequency histogram of residuals (right) between the data and best-fit model for *Eunicea flexuosa*. The color scale indicates how many residuals occur for each combination of Deep (vertical axis) and Shallow (horizontal) ecotype alleles.

**Figure S2.** (top) 2D allele frequency spectra from *Agaricia fragilis* for the data (left) and the model (right). The color scale indicates how many SNPs occur for each combination of Deep (D) and Shallow (S) ecotype alleles. (bottom) The 2D frequency spectrum of residuals (left) and frequency histogram of residuals (right) between the data and best-fit model for *Agaricia fragilis*.

**Figure S3.** (top) 2D allele frequency spectra from *Pocillopora damicornis* for the data (left) and the model (right). The color scale indicates how many SNPs occur for each combination of Reef Flat (F) and Slope (S) ecotype alleles. (bottom) The 2D frequency spectrum of residuals (left) and frequency histogram of residuals (right) between the data and best-fit model for *Pocillopora damicornis*.
